# Association of Clinical Features of Colorectal Cancer with Circulating Tumor Cells and Systemic Inflammatory Markers

**DOI:** 10.1155/2022/5105599

**Published:** 2022-04-21

**Authors:** Yasi Xing, Fangyuan Qin, Yaping Zhai, Jingwen Yang, Yiyang Yan, Dan Li, Han Zhang, Renwang Hu, Xianjing Xu, Xuanchao Cao, Gairong Huang, Xiang Liu

**Affiliations:** ^1^Henan Eye Institute, Henan Eye Hospital, Henan Provincial People's Hospital/People's Hospital of Zhengzhou University, 7 Weiwu Road, Zhengzhou, Henan Province, China 450003; ^2^Department of General Surgery, Henan Provincial People's Hospital/People's Hospital of Zhengzhou University, 7 Weiwu Road, Zhengzhou, Henan Province, China 450003; ^3^Department of Geriatric Medicine, Henan Provincial People's Hospital/People's Hospital of Zhengzhou University, 7 Weiwu Road, Zhengzhou, Henan Province, China 450003

## Abstract

**Background:**

Circulating tumor cells (CTCs) in peripheral blood have been shown to reflect the prognosis of patients with colorectal cancer, and epithelial and mesenchymal markers further predict the likelihood of cancer dissemination. This study was conducted to identify possible association of clinical features of colorectal cancer with CTC counts, their subtypes, and systemic inflammatory markers.

**Methods:**

Blood samples of 316 colorectal cancer patients were used for CTC detection and subtyping with EpCAM, CK8/18/19, vimentin, and twist as biomarkers. The neutrophil/lymphocyte ratio, platelet/lymphocyte ratio, C-reactive protein/albumin ratio, lymphocyte/monocyte ratio, and systemic immune-inflammation index (SII) were also measured. The relationship between clinical data and these markers or parameters was analyzed.

**Results:**

Total CTC counts were correlated with whether there was lymph node involvement but was not correlated with TNM staging. There was a difference in mesenchymal CTCs between patients with and without lymph node involvement (*P* < 0.05). Also, more patients with metastasis tested positive for mesenchymal CTCs (*P* < 0.05). Of the systemic inflammatory markers, platelet/lymphocyte ratio was positively correlated with CTC counts (*P* < 0.01), and lymphocyte/monocyte ratio was negatively correlated with CTC counts (*P* < 0.05).

**Conclusions:**

Colorectal cancer patients with the mesenchymal markers on their CTCs are more likely to have lymph node involvement or distant metastasis than those without these markers.

## 1. Introduction

Colorectal cancer (CRC) is one of the most common malignancies and a leading cause of death. CRC-related mortality is mostly attributable to metastasis to other organs and tissues, especially the liver, lung, and peritoneum [1, 2]. About 20% of patients are found to have metastases at the time of CRC diagnosis, and many more will develop metastasis within five years of tumor resection [1]. Although poor outcomes are generally expected with metastasis, the spread of CRC to some sites, the peritoneum, for example, signifies much shorter overall survival than metastatic CRC in other sites and may possess distinctive phenotypic and genotypic features [3, 4]. Therefore, biomarkers may be needed to provide an alternative for the assessment of prognosis to the most widely used TNM staging system, which does not consider some known risk factors.

The detection of circulating tumor cells (CTCs) in peripheral blood has been used to predict the existence of disseminated tumor cells and the risk of cancer recurrence. Studies have shown that CTCs are able to predict distant metastasis and overall survival in several types of cancer [5, 6]. For nonmetastatic CRC, detection of CTCs before surgery was associated with unfavorable outcomes [7, 8]. In patients with metastatic CRC, higher counts of CTCs before and during treatment were an independent predictor of progression-free and overall survival [9, 10]. However, results from other studies did not support a prognostic role for CTCs in CRC [11, 12]. The conflicting findings might have been due to discrepancies in patient characteristics and detection protocols between these studies, but the site of metastasis also has a major impact on prognosis for CRC. For instance, although epithelial-mesenchymal transition (EMT) is often thought to be a primary mechanism promoting cancer progression and metastasis, CRC metastases in different organs and tissues may not share the same pathways and promoting factors [13].

A recently introduced technology uses epithelial and mesenchymal markers (EpCAM, CK8/18/19, vimentin and twist) to classify CTCs into three subtypes, epithelial CTCs, epithelial/mesenchymal CTCs, and mesenchymal CTCs [14–16]. Yet, it remains unclear whether there is a connection between CTC subpopulations and the risk of CRC metastasis. In this study, we analyzed CTC phenotypes, metastatic patterns, and other clinicopathological features in CRC patients. The association of common systemic inflammatory markers with characteristics of CRC was also evaluated.

## 2. Methods

### 2.1. Participant Recruitment

Subjects were recruited between May 2016 and September 2020. Patients who were histologically diagnosed with colon or rectal cancer and had complete medical records at our hospital were eligible for inclusion. The research protocol was approved by the ethics committee of Henan Provincial People's Hospital. Written informed consent was obtained from participants in accordance with the Declaration of Helsinki. A total of 316 patients entered the study, with general data containing clinical staging, recurrence, sites of metastases, and histological classifications.

### 2.2. Isolation of CTCs and Detection of EMT Biomarkers

Immediately following diagnosis with CRC, 5 ml of the patient's peripheral blood was drawn into a centrifuge tube containing EDTA after discarding the first 2 ml to prevent skin cell contamination. The CTC isolation method used in this study was initially described by Wu et al. [14]. To remove red blood cells, a lysis buffer (154 mM NH_4_Cl, 10 mM KHCO_3_, and 0.1 mM EDTA) (Sigma-Aldrich, St. Louis, USA) was added. After 30 min, centrifugation was performed at 1850 rpm for 5 min, and then the supernatant was extracted. The remaining cells were resuspended in a mixture of 4 ml PBS and 1 ml 4% formaldehyde. The suspension was vortexed and placed at room temperature for 8 min. CTCs were isolated with a filtration system consisting of a filtration tube containing a membrane with a pore diameter of 8 *μ*m, a multiwell plate vacuum manifold, and a vacuum pump with the pressure set at ≥0.08 MPa.

RNA in situ hybridization (RNA-ISH) was used to detect CTC biomarkers, which included epithelial markers EpCAM and CK8/18/19, mesenchymal markers vimentin and twist, and CD45 as a leukocyte marker.  Table 1 shows the sequences of capture probes for CTC biomarker genes, which were all synthesized by Invitrogen (Invitrogen, Shanghai, China). After treatment of the cells on the membranes with a protease (Qiagen, Hilden, Germany), capture probes for the above biomarkers were used for hybridization at 42°C for 2 hours, followed by washing three times with 1000 ml of a wash buffer (0.1× SSC) (Sigma-Aldrich, St. Louis, USA) to remove unbound probes. For signal amplification, samples were incubated at 42°C for 20 minutes with a 100 *μ*l of a preamplifier solution composed of 30% horse serum, 1.5% sodium dodecyl sulfate, 3 mM Tris-HCl (pH 8.0) (Sigma-Aldrich, St. Louis, USA), and 0.5 fmol of the preamplifier. After cooling, the membranes were again washed three times with 0.1× SSC and then incubated with 100 *μ*l of the amplifier solution.

**Figure 1:**
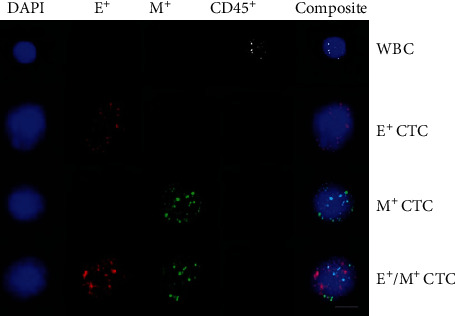
EMT phenotypes of CTCs and white blood cells (WBCs) were detected by RNA in situ hybridization in CRC patients. Cell nuclei were stained with DAPI (blue). White blood cells (WBCs) were detected using CD45 as the marker (white). Epithelial circulating tumor cells (E^+^ CTCs) were detected using EpCAM and CK8/18/19 as markers (red); mesenchymal circulating tumor cells (M^+^ CTCs) were detected with vimentin and twist as markers (green) dots; E^+^/M^+^ CTCs exhibited both epithelial and mesenchymal markers (red and green) (Under ×10 eyepiece and ×40 objective magnification. Scale bar = 5 *μ*m).

Three fluorescent dyes were used to label the probes, with Alexa Fluor 594 for the EpCAM and CK8/18/19 probes, Alexa Fluor 488 for the vimentin and twist probes, and Alexa Fluor 647 for the CD45 probe. After washing with 0.1× SSC, the cells were stained with 4′,6-diamidino-2-phenylindole (DAPI) (Sigma, St. Louis, USA) for 5 minutes and examined with an Axio Imager Z2 fluorescence microscope (ZEISS, Oberkochen, Germany). After filtration, CTC counts were obtained with the subtraction of CD45^+^ cells. CD45^+^ cells were labeled with Alexa Fluor 647 (white fluorescence), epithelial markers (EpCAM and CK8/18/19) were labeled with Alexa Fluor 594 (red fluorescence), and mesenchymal markers (vimentin and twist) were labeled with Alexa Fluor 488 (green fluorescence). Cells possessing epithelial and mesenchymal markers (E + M) showed both red and green fluorescence. Images of white blood cells, epithelial CTCs, mesenchymal CTCs, and epithelial/mesenchymal CTCs are shown in Figure 1.

### 2.3. Clinical Laboratory Tests

Patients' blood samples were sent to the hospital's clinical laboratories for the detection of carcinoembryonic antigen (CEA) and cancer antigen 19-9 (CA 19-9). Commercial electrochemical luminescence kits (Roche-Diagnostics, Shanghai, China) were used for these markers. Tests were conducted according to the manufacturer's instructions. Laboratory results for the neutrophil/lymphocyte ratio (NLR), platelet/lymphocyte ratio (PLR), C-reactive protein/albumin ratio (CAR), lymphocyte/monocyte ratio (LMR), and systemic immune-inflammation index (SII) were also collected.

### 2.4. Statistical Analysis

All statistical analyses were conducted using SPSS, version 18.0 (SPSS Inc., 154 Chicago, IL), and significance was set at .05 for all tests. For laboratory parameters, common reference values were used to categorize patients. Correlations between variables were examined using the two-sided *χ*^2^ test or the *t*-test, where applicable.

## 3. Results

A total of 316 CRC patients were included in this study. Demographic data, clinical features, and histological results are listed in Table 2. Of note, 46.8% of patients were classified as TNM stages III and IV, indicating various degrees of lymph node involvement. There were 62 patients with distant metastasis (stage IV), with 16 cases to the liver, 10 to the lungs, 13 to the peritoneum, 7 to the bone, and 16 to multiple sites.

**Figure 2:**
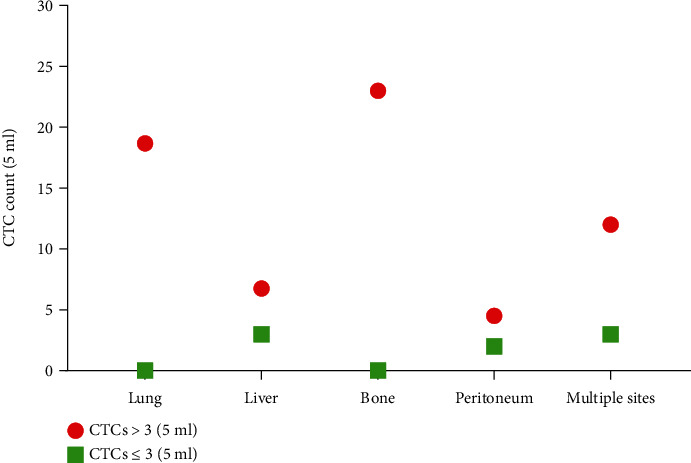
CTC counts in patients (*n* = 62) with different metastasis sites, including the lung (*n* = 10), liver (*n* = 16), bone (*n* = 7), peritoneum (*n* = 13), and multiple sites (*n* = 16). Each icon represents the average CTC count per patient. For each site of metastasis, most patients had CTC counts > 3/5 ml.

Using CTC counts ≤ 3 as negative detection and >3 as positive detection, we found that total CTC counts were correlated with whether there was lymph node involvement but were not correlated with TNM staging. In addition, there was no relationship between total CTC counts and any of the features such as history of smoking, tumor size, or histological grade (Table 3). As for CTC counts in patients with different sites of metastasis, it seemed that metastasis to each site was accompanied with higher numbers of patients with positive CTC detection than with negative CTC detection (Figure 2). However, since the numbers in the groups were very small, we did not conduct any statistical analysis to see if there were differences in CTC detection rates between these groups.

**Figure 3:**
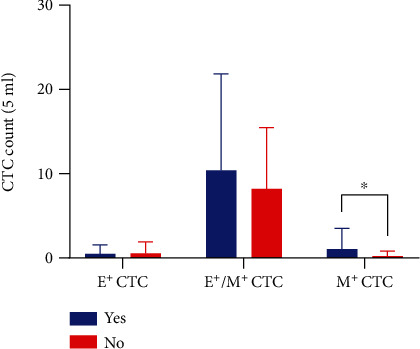
CTC subtypes in CRC patients with (blue) or without (red) lymph node involvement. Patients with lymph node involvement had significantly higher M^+^ CTC counts than those without (^∗^*P* < 0.05), but there was no difference in E^+^ or E^+^/M^+^ CTC counts between the two groups.

**Figure 4:**
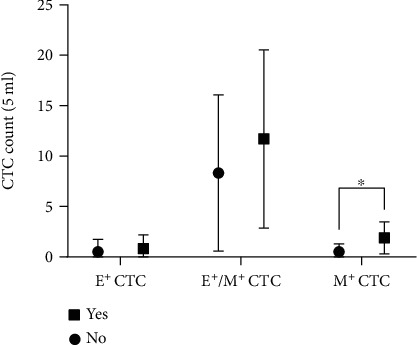
CTC subtypes in CRC patients with (stage IV) or without (stages I-III) distant metastasis. Patients with distant metastasis had significantly higher M^+^ CTC counts than patients without distant metastasis (^∗^*P* < 0.05).

Subsequent analyses were aimed to identify any potential association of lymph node involvement or metastasis with CTC subtypes. To determine whether CTC subtypes were correlated with lymph node involvement or remote metastasis, we first divided patients into two groups, one with lymph node involvement and the other without, regardless of tumor staging. Our analysis showed that there was a difference in mesenchymal CTCs (*P* < 0.05) while no difference was found in the epithelial phenotype or the mixed phenotype between these two patient groups (Figure 3). Then, we compared CTC subtype counts between patients with remote metastasis (stage IV) and those without (stages I-III) and found that there was a difference in mesenchymal CTCs but not in epithelial or mixed CTCs between these patients (*P* < 0.05) (Figure 4).

More CRC patients had CEA and CA 19-9 levels within the reference ranges (≤ 3.5 ng/ml for CEA and ≤30 U/ml for CA 19-9) than those with CEA and CA 19-9 levels exceeding the reference ranges. Also, the levels of these two markers were not associated with CTC counts (Table 4). Of the five systemic inflammatory markers, PLR was positively correlated with CTC counts (*P* < 0.01), and LMR was negatively correlated with CTC counts (*P* < 0.05), whereas none of NLR, CAR, and SII showed any correlation with CTC counts (Table 4).

## 4. Discussion

One of the major findings in the current study is that total CTC counts were positively correlated with lymph node involvement but not with TNM staging in CRC patients. We were unable to identify any relationship between CTC subtypes and specific sites of metastasis. A number of studies have demonstrated that CTCs may serve as a prognostic tool for CRC [5, 6]. Around the time of diagnosis, patients with CTCs detected in peripheral blood face unfavorable outcomes, compared with those without detectable CTCs [7, 8]. It is assumed that a significant portion of patients who have undergone curative tumor resection still retain clinically undetectable micrometastases or minimal residual disease, which may enter the circulation and become the sources of later metastasis or recurrence [17, 18]. However, very limited research has been conducted on possible links between CTC subtypes and clinicopathological features in CRC. Two studies using CTC detection methods similar to ours have found that both total CTCs and mesenchymal CTCs are closely associated with lymph node involvement and CRC metastasis than other subtypes of CTCs [15, 16]. Our results suggest that mesenchymal CTCs are a more sensitive to predict lymph node involvement or distant metastasis. Probably as a result of relatively small sample sizes, neither of the two studies analyzed the relationship between CTC counts and metastatic patterns. Although we had a much larger sample, the number of cases for each site of metastasis was still low. Given that other factors may obscure the impact of CTC counts, if any, this issue remains unsettled.

The demonstration in our study that patients with higher counts of mesenchymal CTCs were more likely to have remote metastasis further illustrates the important role EMT plays in CRC dissemination. During EMT, epithelial tumor cells acquire a mesenchymal phenotype through a highly coordinated program that involves extracellular signals such as multiple growth factors and cytokines, transcription factors of genes responsible for cell adhesion and mesenchymal differentiation, and consequently molecules that confer a distinctive epithelial or mesenchymal identity to the cells [19, 20]. We used several common biomarkers for CTC subtyping to identify epithelial or mesenchymal features. EpCAM is often overexpressed in epithelial tumor cells and interacts with cell adhesion molecules including E-cadherin and claudins [21, 22]. It is known to regulate cell growth via the Wnt and TGF-*β* signaling pathways [23]. Ck8/18/19 are also abundant markers present in epithelial cancers [24, 25]. Vimentin is a major component of the intermediate filaments, and its expression leads to increased cellular migration and invasion [26, 27]. As a transcription factor, TWIST promotes the downregulation of E-cadherin and upregulation of mesenchymal markers such as fibronectin, N-cadherin, and vimentin [28]. Its overexpression is associated with poor prognosis in cancer patients [29, 30]. Of course, CTC counts and phenotypes alone are not sufficient to determine whether tumor cells will achieve successful colonization in distant organs, which requires evasion of immune defenses and adaptation to the tumor microenvironment.

In addition to biological processes required for remote metastasis such as EMT, factors that influence the route and site of CRC metastasis remain poorly understood. EMT promotes the development of mesenchymal features in tumor cells. However, CTCs may also undergo mesenchymal-to-epithelial transition (MET), the reverse of EMT, to colonize metastatic sites [31, 32]. The liver and peritoneum are among the most common sites of CRC metastasis, but the pathways to these organs may be different [33]. Metastasis to the liver is thought to follow the typical invasion-metastasis cascade, whereby primary tumor cells first invade the surrounding local tissues and then intravasate into the circulation [34]. After transporting to the distant organ site, they extravasate into the parenchyma of the organ, adapt to the new environment, and resume proliferation. In contrast, the spread to the peritoneum includes detachment of primary tumor cells, local migration, adhering to the peritoneal surface, infiltration into deep layers, and proliferation [33, 35]. With either pathway, tumor cells need to adopt EMT-related changes to survive the processes. In order to examine whether CTC counts could indicate a predilection for metastasis to specific organs, we divided the patients into five groups based on the site of metastasis. Although there were more CTC-positive cases than CTC-negative cases in each group, the numbers of CTC-negative cases were too small to allow meaningful statistical analysis. Therefore, the value of CTCs in assessing organ-specific metastasis needs to be further investigated.

Since inflammation has been suggested to play a critical role in the development and metastasis of many types of cancer, we included several systemic inflammatory parameters and analyzed their potential association with CTCs in this study. Our results showed that CTC counts were positively correlated with PLR and negatively correlated with LMR but had no correlation with NLR, CAR, or SII. These ratios reflect different aspects of interaction between immune and inflammatory factors in response to tumor development. Platelets suppress lymphocyte proliferation through P-selectin, resulting in decreased proinflammatory cytokine levels and increased anti-inflammatory cytokine levels; so, its positive correlation with CTCs should be expected [36, 37]. In addition, the lymphocyte count indicates antitumor immunity while monocytes, especially those that differentiate into tumor-associated macrophages, are considered to favor tumor growth and metastasis [38]. Therefore, a high LMR is also consistent with a high CTC count. On the other hand, although many studies have reported strong associations of a high NLR and a high CAR with adverse survival and of high SII levels with improved outcomes for cancer patients, including those with CRC [39–41], we were unable to confirm these findings. As pointed out by others, the overall data supporting these systemic inflammatory parameters as strong prognostic indicators for CRC are less than convincing, probably due to a lack of a clear understanding of the immune and inflammatory processes underlying tumor pathophysiology and discrepancies in research designs and evaluation criteria employed by different studies [42].

There were some limitations in our study. As a convenient and minimally invasive technology, CTC detection has proved to be a viable alternative biopsy option, but the method we used is based on mRNAs of epithelial and mesenchymal markers and may not reflect protein expression. Moreover, aneuploidy of chromosomes, especially 7 and 8, is often seen in CRC [43, 44]. An integrated approach, which comprises subtraction enrichment and immunostaining-fluorescence in situ hybridization and takes into account of cell morphology, proteins, and nucleic acids, has been introduced to maximize the accuracy of CTC detection [45]. Also, although our study confirmed that patients with high total CTC counts or with high mesenchymal CTCs were more likely to have lymph node involvement and was among the very few with relatively large samples of CRC patients, its cross-sectional design prevented us from accurately estimating the predictive value of CTCs for CRC outcomes. Other studies have used CTC detection to monitor survival and recurrence and to examine the efficacy of treatment regimens, but differences in the time of sample collection, choice of methods, follow-up lengths, and cut-off values have produced inconsistent results. Hopefully, further research may overcome these deficiencies and also consider combining other biomarkers to realize more accurate assessment of prognosis and therapeutic efficacy.

In conclusion, available studies, including ours, have demonstrated that CTC detection may provide a convenient tool for the diagnosis and treatment planning of CRC. Subtyping of CTCs is an especially promising approach to the prediction of clinical outcomes because of the increasingly recognized role of EMT in tumor dissemination. For a better understanding of the value of CTC detection and subtyping in CRC management, more rigorous evidence needs to be obtained, preferably through improved research designs, standardized detection protocols, and follow-up of large cohorts.

## Figures and Tables

**Table 1:** Sequences of capture probes for CTC biomarker genes.

Gene	Sequences (5′→3′)
EpCAM	TGGTGCTCGTTGATGAGTCAAGCCAGCTTTGAGCAAATGA AAAGCCCATCATTGTTCTGGCTCTCATCGCAGTCAGGATC TCCTTGTCTGTTCTTCTGACCTCAGAGCAGGTTATTTCAG
CK8	CGTACCTTGTCTATGAAGGAACTTGGTCTCCAGCATCTTG CCTAAGGTTGTTGATGTAGCCTGAGGAAGTTGATCTCGTC CAGATGTGTCCGAGATCTGGTGACCTCAGCAATGATGCTG
CK18	AGAAAGGACAGGACTCAGGCGAGTGGTGAAGCTCATGCTG TCAGGTCCTCGATGATCTTGCAATCTGCAGAACGATGCGA AGTCATCAGCAGCAAGACGCTGCAGTCGTGTGATATTGG
CK19	CTGTAGGAAGTCATGGCGAGAAGTCATCTGCAGCCAGACG CTGTTCCGTCTCAAACTTGGTTCTTCTTCAGGTAGGCCAGC TCAGCGTACTGATTTCCTCGTGAACCAGGCTTCAGCATC
Vimentin	GAGCGAGAGTGGCAGAGGACCTTTGTCGTTGGTTAGCTGG CATATTGCTGACGTACGTCAGAGCGCCCCTAAGTTTTTAAA GATTGCAGGGTGTTTTCGGGCCAATAGTGTCTTGGTAG
Twist	ACAATGACATCTAGGTCTCCCTGGTAGAGGAAGTCGATGTC AACTGTTCAGACTTCTATCCCTCTTGAGAATGCATGCATTTTC AGTGGCTGATTGGCACTTACCATGGGTCCTCAATAA
CD45	TCGCAATTCTTATGCGACTCTGTCATGGAGACAGTCATGTG TATTTCCAGCTTCAACTTCCCATCAATATAGCTGGCATTTTG TGCAGCAATGTATTTCCTACTTGAACCATCAGGCATC

**Table 2:** Baseline patients characteristics (*n* = 316).

Parameter	Number of patients (%)
Sex	
Male	202 (63.9%)
Female	114 (36.1%)
Age	
≤60	196 (62.0%)
>60	120 (38.0%)
Smoking	
Yes	148 (46.8%)
No	168 (53.2%)
Tumor size	
≤3 cm	110 (34.8%)
**>**3 cm	206 (65.2%)
Differentiation	
Poor	112 (35.4%)
Well and moderate	204 (64.6%)
Lymph node metastasis	
Yes	108 (34.2%)
No	208 (65.8%)
TNM stage	
I	58 (18.4%)
II	110 (34.8%)
III	86 (27.2%)
IV	62 (19.6%)

**Table 3:** Correlation between CTC detection and clinical and histological parameters.

Parameter	Number of patients with **>**3	CTCs ≤ 3	*P* value	*R*	*χ* ^2^
Smoking			0.19	——	1.69
Yes	120	28			
No	126	42			
Tumor size			0.29	——	1.14
≤3 cm	92	18			
>3 cm	162	44			
Differentiation			0.99	——	0.001
Poor	90	22			
Well and moderate	164	40			
Lymph node involvement			0.01^∗^	0.15	7
Yes	87	21			
No	138	70			
TNM stage			0.57	——	2.00
I	48	10			
II	86	24			
III	64	22			
IV	51	11			

^∗^ indicates that the correlation was statistically significant. Only lymph node involvement was correlated with positive CTC detection.

**Table 4:** Correlation between CTC detection and systemic inflammatory markers.

Parameter	Number of patients with >3	CTCs ≤ 3	*P* value	*R*	*χ* ^2^
CEA			0.4	——	0.72
≤3.5 ng/ml	178	40			
>3.5 ng/m	76	22			
CA 19-9			0.13	——	2.29
≤30 U/ml	190	52			
>30 U/ml	64	10			
NLR			0.49	——	0.49
≤2.3	160	42			
>2.3	94	20			
PLR			0.001^∗^	0.21	14.4
≤132.5	142	18			
>132.5	112	44			
CAR			0.49	——	0.47
≤0.2	214	50			
>0.2	40	12			
LMR			0.004^∗^	-0.16	8.34
≤4.4	102	44			
>4.4	142	28			
SII			0.09	——	2.94
≤372.9	128	46			
>372.9	116	26			

^∗^ indicates that the correlation was statistically significant. PLR showed a positive correlation (*P* < 0.01), and LMR showed a negative correlation (*P* < 0.05) with positive CTC detection.

## Data Availability

The data presented in this study are available upon request to the corresponding author.

## Abbreviations

CRC: Colorectal cancer
CTC: Circulating tumor cell
EMT: Epithelia-mesenchymal transition
CEA: Carcinoembryonic antigen
CA 19-9: Cancer antigen 19-9
NLR: Neutrophil/lymphocyte ratio
PLR: Platelet/lymphocyte ratio
CAR: C-reactive protein/albumin ratio
LMR: Lymphocyte/monocyte ratio
SII: Systemic immune-inflammation index.

## References

[1] Biller L. H., Schrag D. (2021). Diagnosis and treatment of metastatic colorectal Cancer. *Journal of the American Medical Association*.

[2] Vassos N., Piso P. (2018). Metastatic colorectal cancer to the peritoneum: current treatment options. *Current Treatment Options in Oncology*.

[3] Dekker E., Tanis P. J., Vleugels J. L. A., Kasi P. M., Wallace M. B. (2019). Colorectal cancer. *Lancet*.

[4] Zhu X., Zhou G., Ni P. (2021). CD31 and D2-40 contribute to peritoneal metastasis of colorectal cancer by promoting epithelial-mesenchymal transition. *Gut Liver*.

[5] Heitzer E., Auer M., Ulz P., Geigl J. B., Speicher M. R. (2013). Circulating tumor cells and DNA as liquid biopsies. *Genome Medicine*.

[6] Pantel K., Alix-Panabières C. (2019). Liquid biopsy and minimal residual disease -- latest advances and implications for cure. *Nature Reviews. Clinical Oncology*.

[7] Bork U., Rahbari N. N., Schölch S. (2015). Circulating tumour cells and outcome in non-metastatic colorectal cancer: a prospective study. *British Journal of Cancer*.

[8] Abdalla T. S. A., Meiners J., Riethdorf S. (2021). Prognostic value of preoperative circulating tumor cells counts in patients with UICC stage I-IV colorectal cancer. *PLoS One*.

[9] Salvianti F., Gelmini S., Mancini I. (2021). Circulating tumour cells and cell-free DNA as a prognostic factor in metastatic colorectal cancer: the OMITERC prospective study. *British Journal of Cancer*.

[10] Silva S. E., Abdallah E. A., Mello C. A. L. D., Tariki M. S., Calsavara V. F., Chinen L. T. (2020). Prospective study with circulating tumor cells as potential prognosis biomarker in metastatic colorectal cancer. *Journal of Clinical Oncology*.

[11] Rahbari N. N., Aigner M., Thorlund K. (2010). Meta-analysis shows that detection of circulating tumor cells indicates poor prognosis in patients with colorectal cancer. *Gastroenterology*.

[12] Liberko M., Kolostova K., Bobek V. (2013). Essentials of circulating tumor cells for clinical research and practice. *Critical Reviews in Oncology/Hematology*.

[13] Kim M. J., Lee H. S., Kim J. H. (2012). Different metastatic pattern according to the KRAS mutational status and site-specific discordance of KRAS status in patients with colorectal cancer. *BMC Cancer*.

[14] Wu S., Liu S., Liu Z. (2015). Classification of circulating tumor cells by epithelial-mesenchymal transition markers. *PLoS One*.

[15] Wu F., Zhu J., Mao Y., Li X., Hu B., Zhang D. (2017). Associations between the epithelial-mesenchymal transition phenotypes of circulating tumor cells and the clinicopathological features of patients with colorectal cancer. *Disease Markers*.

[16] Hou J., Guo C., Lyu G. (2020). Clinical significance of epithelial–mesenchymal transition typing of circulating tumour cells in colorectal cancer. *Colorectal Disease*.

[17] Feezor R. J., Copeland E. M., Hochwald S. N. (2002). Significance of micrometastases in colorectal cancer. *Annals of Surgical Oncology*.

[18] Koyanagi K., Bilchik A. J., Saha S. (2008). Prognostic relevance of occult nodal micrometastases and circulating tumor cells in colorectal cancer in a prospective multicenter trial. *Clinical Cancer Research*.

[19] Dongre A., Weinberg R. A. (2019). New insights into the mechanisms of epithelial-mesenchymal transition and implications for cancer. *Nature Reviews. Molecular Cell Biology*.

[20] Zhang N., Ng A. S., Cai S., Li Q., Yang L., Kerr D. (2021). Novel therapeutic strategies: targeting epithelial-mesenchymal transition in colorectal cancer. *The Lancet Oncology*.

[21] Martowicz A., Seeber A., Untergasser G. (2016). The role of EpCAM in physiology and pathology of the epithelium. *Histology and Histopathology*.

[22] Warneke V. S., Behrens H. M., Haag J. (2013). Members of the EpCAM signalling pathway are expressed in gastric cancer tissue and are correlated with patient prognosis. *British Journal of Cancer*.

[23] Karabicici M., Azbazdar Y., Ozhan G., Senturk S., Firtina Karagonlar Z., Erdal E. (2021). Changes in Wnt and TGF-*β* signaling mediate the development of regorafenib resistance in hepatocellular carcinoma cell line HuH7. *Frontiers in Cell and Development Biology*.

[24] Shao M. M., Chan S. K., Yu A. M. (2012). Keratin expression in breast cancers. *Virchows Archiv*.

[25] Masai K., Nakagawa K., Yoshida A. (2014). Cytokeratin 19 expression in primary thoracic tumors and lymph node metastases. *Lung Cancer*.

[26] Vuoriluoto K., Haugen H., Kiviluoto S. (2011). Vimentin regulates EMT induction by slug and oncogenic H-Ras and migration by governing Axl expression in breast cancer. *Oncogene*.

[27] Leduc C., Etienne-Manneville S. (2015). Intermediate filaments in cell migration and invasion: the unusual suspects. *Current Opinion in Cell Biology*.

[28] De S., Das S., Mukherjee S., Das S., Sengupta S. (2017). Establishment of twist-1 and TGFBR2 as direct targets of microRNA-20a in mesenchymal to epithelial transition of breast cancer cell-line MDA-MB-231. *Experimental Cell Research*.

[29] Zhang Y. Q., Chen W. L., Zhang F. (2019). Over-expression of both VEGF-C and twist predicts poor prognosis in human breast cancer. *Clinical & Translational Oncology*.

[30] da Silva S. D., Alaoui-Jamali M. A., Soares F. A. (2014). TWIST1 is a molecular marker for a poor prognosis in oral cancer and represents a potential therapeutic target. *Cancer*.

[31] Chao Y., Wu Q., Acquafondata M., Dhir R., Wells A. (2012). Partial mesenchymal to epithelial reverting transition in breast and prostate cancer metastases. *Cancer Microenvironment*.

[32] Hamilton G., Rath B. (2017). Mesenchymal-epithelial transition and circulating tumor cells in small cell lung cancer. *Advances in Experimental Medicine and Biology*.

[33] Pretzsch E., Bösch F., Neumann J. (2019). Mechanisms of metastasis in colorectal cancer and metastatic organotropism: hematogenous versus peritoneal spread. *Journal of Oncology*.

[34] Bruin S. C., He Y., Mikolajewska-Hanclich I. (2011). Molecular alterations associated with liver metastases development in colorectal cancer patients. *British Journal of Cancer*.

[35] Kranenburg O., van der Speeten K., de Hingh I. (2021). Peritoneal metastases from colorectal cancer: defining and addressing the challenges. *Frontiers in Oncology*.

[36] Dinkla S., van Cranenbroek B., van der Heijden W. A. (2016). Platelet microparticles inhibit IL-17 production by regulatory T cells through P-selectin. *Blood*.

[37] Chen Y., Zhong H., Zhao Y., Luo X., Gao W. (2020). Role of platelet biomarkers in inflammatory response. *Biomarker Research*.

[38] Stefaniuk P., Szymczyk A., Podhorecka M. (2020). The neutrophil to lymphocyte and lymphocyte to monocyte ratios as new prognostic factors in hematological malignancies - a narrative review. *Cancer Management and Research*.

[39] Ethier J. L., Desautels D., Templeton A., Shah P. S., Amir E. (2017). Prognostic role of neutrophil-to-lymphocyte ratio in breast cancer: a systematic review and meta-analysis. *Breast Cancer Research*.

[40] Balde A. I., Fang S., He L. (2017). Propensity score analysis of recurrence for neutrophil-to-lymphocyte ratio in colorectal cancer. *The Journal of Surgical Research*.

[41] Zhou Q. P., Li X. J. (2019). C-reactive protein to albumin ratio in colorectal cancer: a meta-analysis of prognostic value. *Dose Response*.

[42] Rossi S., Basso M., Strippoli A. (2017). Are markers of systemic inflammation good prognostic indicators in colorectal cancer?. *Clinical Colorectal Cancer*.

[43] Ly P., Kim S., Kaisani A., Marian G., Wright W. E., Shay J. W. (2013). Aneuploid human colonic epithelial cells are sensitive to AICAR-induced growth inhibition through EGFR degradation. *Oncogene*.

[44] Wan J. F., Li X. Q., Zhang J. (2018). Aneuploidy of chromosome 8 and mutation of circulating tumor cells predict pathologic complete response in the treatment of locally advanced rectal cancer. *Oncology Letters*.

[45] Lin P. P. (2018). Aneuploid CTC and CEC. *Diagnostics*.

